# Excess mortality associated with HIV: Survey estimates from the PHIA project

**DOI:** 10.4054/demres.2024.51.38

**Published:** 2024-11

**Authors:** Shannon M. Farley, Giles Reid, Kay Yuengling, Connor Wright, Vesper H. Chisumpa, George Bello, James M. Juma, Abigail R. Greenleaf, Stephen McCracken, Paul Stupp, Stéphane Helleringer, Jessica Justman

**Affiliations:** 1 ICAP at Columbia University, New York, NY, USA.; 2 Department of Population Studies, University of Zambia, Lusaka, Zambia.; 3 International Training and Education Centre for Health, Ministry of Health, Lilongwe, Malawi.; 4 National AIDS Control Program, Ministry of Health, Dodoma, Tanzania.; 5 Centers for Disease Control and Prevention, Atlanta, GA, USA.; 6 NYU Abu Dhabi, United Arab Emirates.

## Abstract

**BACKGROUND:**

Incomplete vital statistics systems in resource-limited countries hinder accurate HIV epidemic assessments. Population-based survey data combined with HIV infection biomarkers may partially address this gap, providing excess mortality estimates in households where people living with HIV (PLWH) reside.

**OBJECTIVE:**

Examine household-level excess HIV mortality in households with PLWH using population-based survey data, including mortality reported by heads of households, and HIV biomarkers.

**METHODS:**

We compared mortality between households with and without PLWH using publicly available data from 11 Population-based HIV Impact Assessments conducted between 2015 and 2019 in Cameroon, Côte d’Ivoire, Eswatini, Kenya, Malawi, Namibia, Rwanda, Tanzania, Uganda, Zambia, and Zimbabwe. Eligible, consenting household members provided blood for HIV testing. Household-level regression models estimated deaths per 1,000 person-years (PY) for the three-year period before the survey; death rate ratios were calculated. Quasi-Poisson distribution accounted for household death over-dispersion.

**RESULTS:**

Country-specific deaths rates per 1,000 PY were significantly higher among rural versus urban households for five countries. For example, in Cameroon, the rates were 9.3 (95% confidence interval [CI]: 8.7–9.9) versus 6.5 (95% CI: 5.9–7.1). In six countries, death rates were significantly higher (1.3–1.7-fold) among households with PLWH versus those without. Death rate ratios were significantly higher among rural (1.4–1.8-fold) and urban households (1.6–2.3-fold) with PLWH versus those without in four and three countries, respectively.

**CONCLUSIONS:**

General population household survey findings in multiple countries in Africa indicate that households where PLWH resided experienced excess mortality relative to other households.

**CONTRIBUTION:**

The novel approach we use to describe HIV-related household-level mortality offers an additional method to measure progress toward zero AIDS-related deaths.

## 1. Introduction

Assessing the global HIV epidemic and benchmarking progress requires accurate national estimates of both new HIV infections and HIV-related mortality. AIDS-related deaths have fallen by almost 70% since their peak in 2004, and new infections are at the lowest they have been since the 1980s (UNAIDS 2023). Estimation of HIV-related mortality has received far less attention than new HIV infection rates, however, reflecting challenges with estimating overall mortality in settings with weak and incomplete vital statistics systems (Kharsany and Karim 2016; Mikkelsen et al. 2015). Three mortality-related metrics used to measure progress to zero AIDS deaths include an absolute rate of AIDS-related deaths, the percentage reduction in AIDS-related deaths, and an incidence: mortality ratio (Ghys et al. 2018). In places where vital statistics are not available, models based on other data are used to estimate HIV mortality (Johnson et al. 2019; UNAIDS DATA 2022).

The Spectrum software package is used by national HIV programs to generate annual HIV mortality estimates for 170 countries (UNAIDS DATA 2022). Spectrum models for sub-Saharan Africa (SSA) use mortality data from population cohort data to inform estimates of natural survival in the absence of antiretroviral therapy (ART). Mortality among people on ART is primarily informed by mortality from clinical cohort studies adjusted for under-ascertainment of mortality (Johnson et al. 2019; UNAIDS Indicator Registry 2024).

The model results show how achieving different levels of program coverage in the HIV care continuum impacts global target achievements. For example, if program targets for treatment and viral load suppression are achieved by 2025, global AIDS-related deaths are projected to decrease by 78% (Stover et al. 2021). While these widely used models count deaths directly attributable to HIV, when accounting for non-AIDS mortality among people living with HIV (PLWH), they assume the same non-mortality rates as the total population. This does not account for indirect effects of HIV on mortality, including among those not infected, due to factors such as lower household resources, higher levels of stigma, and other barriers to health care access (IeDEA Pediatric Working Group 2017).

New methods are needed to better document the total effects of HIV on mortality. We propose that biomarkers of HIV infection collected in household surveys offer an important new resource for mortality estimates. Household (HH) surveys, including Demographic and Health Surveys and Multiple Indicator Cluster Surveys, often collect mortality-related data, for example through birth histories, orphanhood data, sibling survival histories, questionnaires about recent household deaths, or assessments of survival in social networks (Stover et al. 2021; IeDEA Pediatric Working Group 2017; Masquelier, Reniers, and Pison 2014; Musarandega et al. 2022). These surveys document all-cause mortality or very specific circumstances of death – for example, those related to pregnancy and childbirth – but do not measure indirect effects of HIV on mortality (Timæus and Nunn 1997; Helleringer et al. 2014; Timæus 1991; Feehan, Mahy, and Salganik 2017). Similarly, Health and Demographic Surveillance Study data are available, but they are subnational, site-based data that are not representative of an entire country (Mee et al. 2016; Slaymaker et al. 2014).

We use data from the Population-based HIV Impact Assessment (PHIA) on recent household deaths in combination with HIV infection biomarkers to investigate excess mortality associated with HIV at the HH level. Additionally, we examine the differences in excess mortality at the HH level when stratified by the urban/rural status of the HH, as mortality often varies between urban and rural locations (Menashe-Oren and Stecklov 2018), and an “urban penalty” has been found in some countries (Gould 1998; Rice and Rice 2009). Our work provides a unique picture of excess mortality associated with HH-level HIV in 11 of the most affected countries in sub-Saharan Africa.

## 2. Methods

### 2.1 PHIA project overview

PHIA surveys were cross-sectional, HH-based surveys designed to estimate national HIV incidence and other HIV control indicators (Sachathep et al. 2021). PHIAs were two-stage cluster surveys designed to be representative at national and subnational levels (Sachathep et al. 2021). Individuals who slept in the HH during the night prior to the survey were eligible for HIV testing if they also met age-related criteria, which varied by country. In Zambia, individuals from birth to 59 years old were eligible, whereas individuals up to age 64 were eligible in Cameroon, Côte d’Ivoire, Kenya, Malawi, Namibia, Rwanda, and Uganda. In Eswatini, Tanzania, and Zimbabwe, there was no upper age limit. In Côte d’Ivoire, children aged 0–14 with mothers who were deceased, tested HIV positive, or had unknown HIV status were eligible for blood testing, as well as a small random sample of children with mothers who tested negative for HIV. In other countries, children aged 0–15 (10–14 in Rwanda) in randomly selected subsets of households (33%–50%, depending on the country) were eligible for blood draw and HIV testing, subject to child assent and parental consent criteria, which varied by age and country. HIV testing was conducted according to each national HIV testing algorithm, and all sero-reactive blood samples underwent confirmatory HIV testing using harmonized procedures across all surveys (Sachathep et al. 2021). The median number of HH residents was four or five, with eight countries reporting a median of four and three countries reporting a median of five HH members (Ministry of Health and Division of Health Operations Research 2020; Ministère de la Santé et de l’Hygiène Publique 2021; Government of the Kingdom of Eswatini 2019; National AIDS and STI Control Programme 2022; Ministry of Health, Malawi 2018; Ministry of Health and Social Services, Namibia 2019; Rwanda Biomedical Center 2020; Tanzania Commission for AIDS and Zanzibar AIDS Commission 2018; Ministry of Health, Uganda 2019; Ministry of Health, Zambia 2019; Ministry of Health and Child Care, Zimbabwe 2019).

### 2.2 Source of data on mortality

In each HH, the head or another competent informant was asked to provide a list of all usual HH members and to state whether any usual HH members had died in the prior three years. The questions about HH deaths were similar in all 11 PHIA surveys:

- Has any usual resident in/of your household died since January 1, 20XX?
- How many usual household residents died since January 1, 20XX?
- What was the name of the person who died (most recently/before him or her)?
- When did [NAME] die? Please give your best guess.
- Was [NAME] male or female?
- How old was [NAME] when they died?

### 2.3 Statistical analyses

We calculated the number of reported deaths per 1,000 person-years (PY) for each country during the three-year period before the survey, assuming that all HH members had resided there for the full three years and that the deceased had resided in the HH until their date of death. For survivors, PY were computed by calculating the length of time between the interview and reference dates (January 1 of either 2015, 2016, or 2017, depending on the survey). For those who died, PY were calculated as the length of time between the reported date of death and the reference date.

When year of death was missing, we assumed the death occurred halfway between the interview and the reference date. Where year but not month was reported, we assumed the death occurred halfway through the reported year. Individuals who were not usual HH residents were excluded.

We evaluated three methods for household inclusion in the analysis: (1) include all households, inputting “No HIV+ members” if a household had no HIV test results; (2) include only households with complete HIV test results; and (3) include households with at least one HIV test result. Since all three methods produced similar results with minimal differences, we selected the third option, to include all households with one or more valid HIV test result. The effects of recall errors were evaluated by analyzing only the subset of deaths reported to have occurred in the same and previous year of the survey, and we found the results did not substantially differ. We therefore opted to include all reported deaths in the analysis.

We used household-level quasi-Poisson regressions with the number of deaths as the outcome variable and the total PY as the offset. A quasi-Poisson distribution accounted for observed over-dispersion of number of deaths per household. Household survey weights were used to compute estimates, and jackknife replicate weights were used to compute confidence intervals (CIs) for both death rates and death rate ratios using the svyglm function from the survey package in R 4.2.2 (Lumley 2023).

Death rates by age group, gender, or urban/rural residence subpopulations were estimated by stratifying individuals by the corresponding categorical variables and fitting a separate model for each subpopulation stratum. Death rate ratios were calculated by including appropriate categorical variables in the model and computing exponentiated regression coefficients to compare with the reference group.

We measured mortality differences associated with the presence of PLWH in the HH. If a household had one or more HH members who tested HIV positive in the survey, for this analysis the HH was designated as “HH with PLWH.” If the HH had zero HH members who tested HIV positive, the HH was designated as “HH without PLWH.” Households with missing data on HH HIV status were excluded from analysis. Models were fit using the entire multi-country sample as well as by country to check for variations by country.

Descriptive statistics regarding the absolute number of HH deaths reported by location (urban or rural) were examined. Data were weighted with HH weights, which are scaled to the total number of households through the selection probabilities at the enumeration area and HH levels. Country-specific weighted death rates per 1,000 PY were calculated overall and by urban/rural residence. Pooled multi-county household death rates by age group, sex, and residence were examined overall and stratified by households with and without PLWH, as were death rate ratios comparing households with and without PLWH. Deaths for younger age groups, 0–5, were bottom coded due to small numbers. Country-specific death rates for households with and without PLWH were examined. Death rates by country between households with and without PLWH stratified by residence were compared. The ratio of death rates in urban and rural households stratified by households with and without PLWH was estimated. Comparisons between groups were presented when the differences were significant.

## 3. Results

The number of households in each survey ranged from 5,185 in Eswatini to 16,918 in Kenya (Table 1). The total unweighted number of household deaths reported by HH respondents due to any cause in the three years prior to each survey in urban areas varied from 61 in Eswatini to 559 in Cameroon; in rural areas it varied from 391 in Eswatini to 1,208 in Cameroon. The percentage of households with PLWH ranged from 4.9% (95% CI: 4.3–5.5) in Côte d’Ivoire to 44.2% (95% CI: 42.8–45.6) in Eswatini. The percentage of the population that lived in household HH with a PLWH member ranged from 5.8% (95% CI: 5.6–6.1) in Côte d’Ivoire to 49.3% (95% CI: 48.6–50.0) in Eswatini. Overall, death rates were similar across countries, with higher rates among rural households compared with urban households, although rates were significantly higher in only 5 out of 11 countries. Overall death rates, defined as estimated number of deaths per 1,000 PY from any cause among all ages and genders in the prior three years, ranged from 4.0 (95% CI: 3.7–4.3) in Rwanda to 7.9 in both Cameroon and Eswatini (95% CI: 7.5–8.4 and 7.1–8.8, respectively). Death rates among urban households ranged from 3.2 (95% CI: 2.6–3.9) in Rwanda to 6.5 (95% CI: 5.9–7.1) in Cameroon. Death rates among rural households ranged from 4.1 (95% CI: 3.8–4.6) in Rwanda to 9.3 (95% CI: 8.7–9.9) in Cameroon.

We found mortality differences associated with the presence of PLWH in the HH across all countries and for several specific countries. Pooled data from all 11 countries, analyzed by age, gender, and urban/rural residence, demonstrated higher death rates among households with PLWH compared with those without PLWH, with death rate ratios greater than 1 for nearly all demographic categories except under age 5, aged 15–24, and aged 50–64 (Table 2). Death rates for HH members aged 25–34 were twice as high in households with PLWH compared to those without PLWH (3.4 [2.7, 4.3] versus 1.5 [1.3, 1.8]; death rate ratio 2.2).

**Figure 1:**
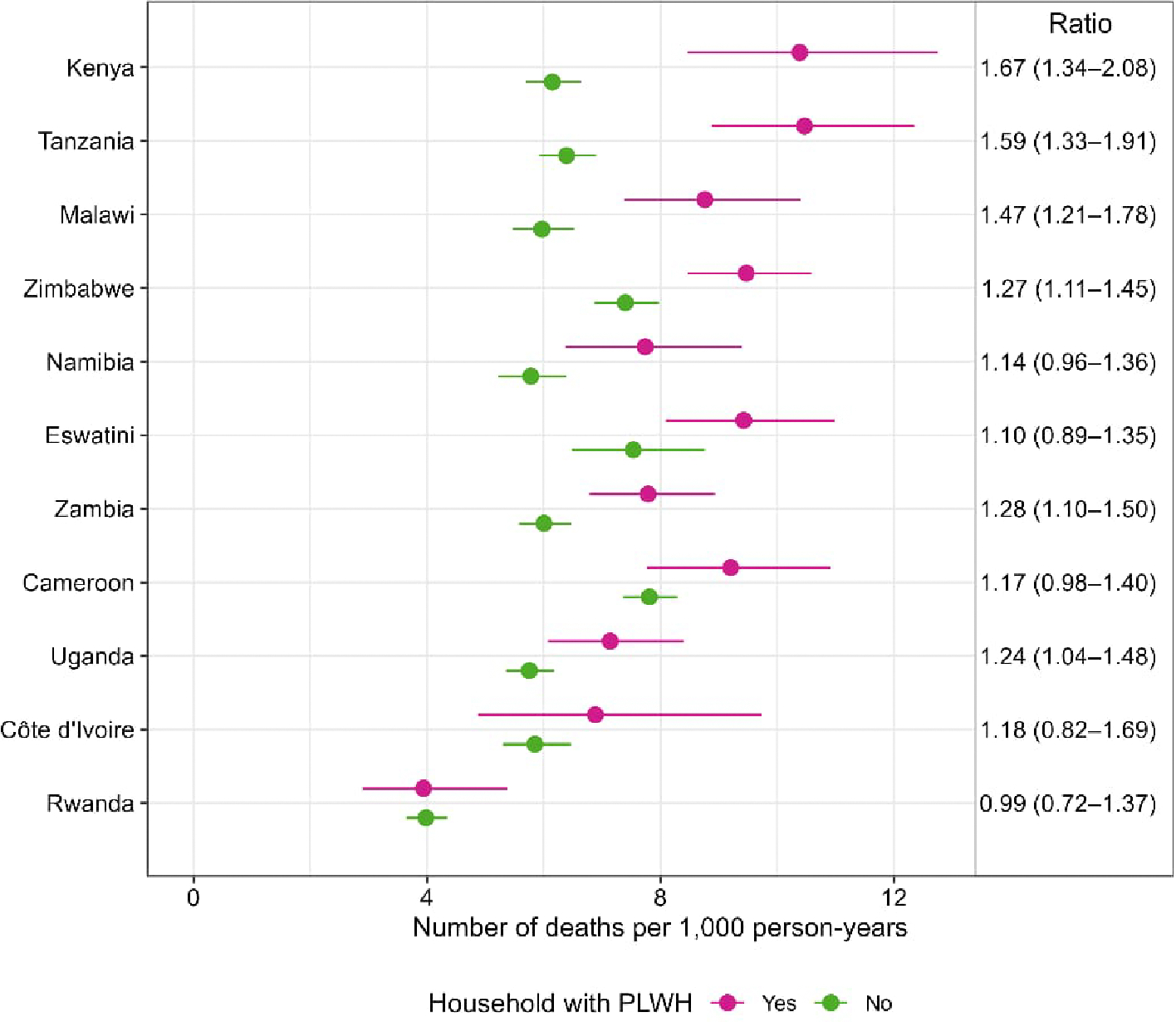
Death rates and death rate ratios by household HIV status including all ages and sexes combined in 11 countries in the three years before the survey, 2015–2019 *Notes*: Countries ordered by magnitude of difference. Death rates among households with and without PLWH are listed as ratios with 95% CI.

Death rates in country-specific models were 1.2–1.7-fold higher among households with PLWH compared to those without PLWH, with death rate ratios greater than 1 for six countries: Kenya (1.67, 95% CI :1.34–2.08), Tanzania (1.59, 95% CI: 1.33–1.91), Malawi (1.47, 95% CI: 1.21–1.78), Zimbabwe (1.27, 95% CI: 1.11–1.45), Zambia (1.28, 95% CI: 1.10–1.50), and Uganda (1.24, 95% CI: 1.04–1.48) (Figure 1). In the remaining five countries, death rates and ratios were not significantly different by HH HIV status.

**Figure 2:**
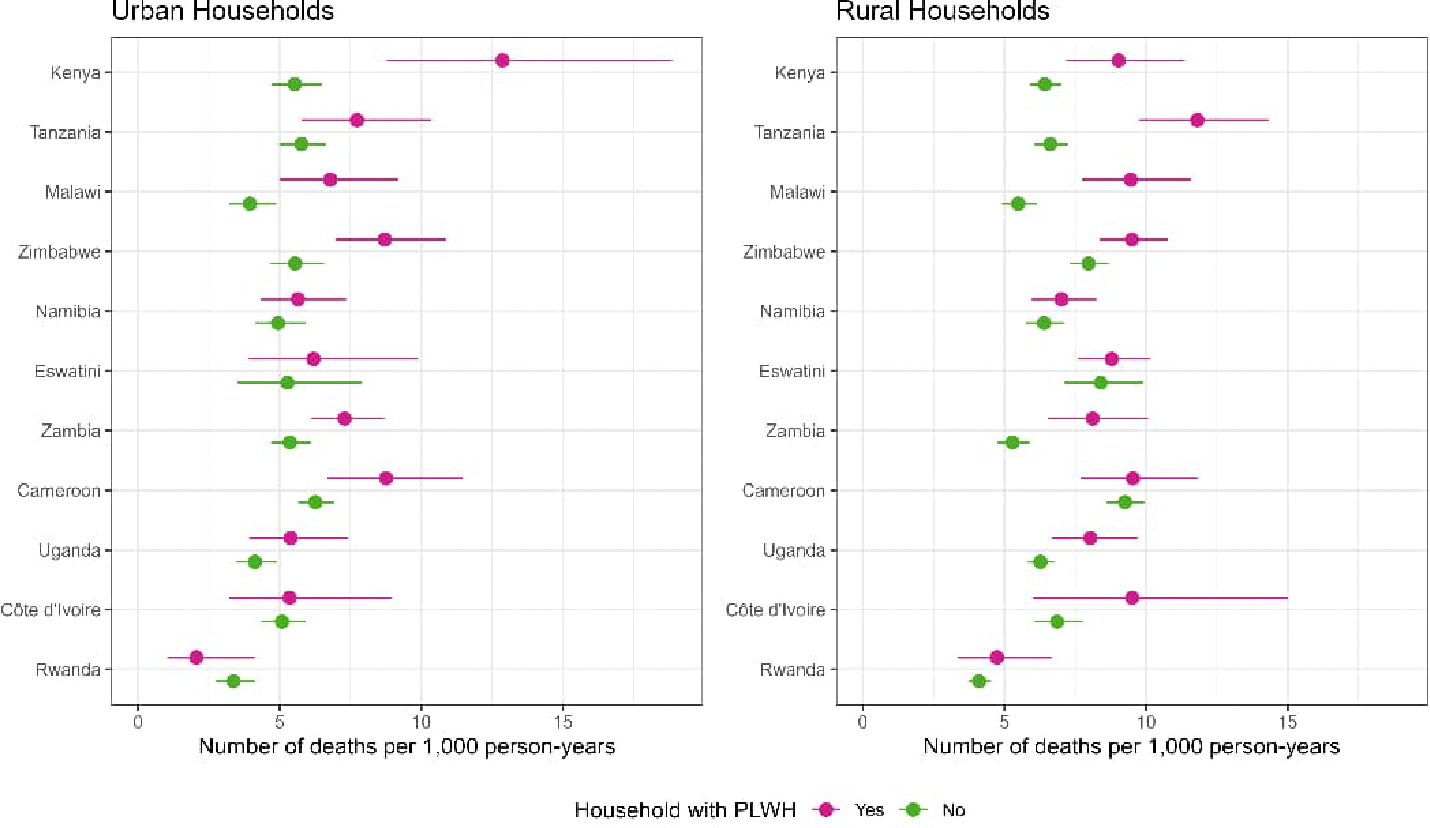
Death rates comparing household HIV status stratified by urban/rural, including all ages and sexes combined in 11 countries in the three years before the survey, 2015–2019 *Note*: Countries ordered by overall magnitude of difference

In separate models for urban and rural households with and without PLWH, we found that urban households with any PLWH had higher death rates per 1,000 PY than urban household without any PLWH in three countries: Kenya (12.9 [95% CI: 8.8–18.9] versus 5.5 [95% CI: 4.7–6.5]), Malawi (6.8 [95% CI: 5.0–9.2] versus 4.0 [95% CI: 3.2–4.9]), and Zimbabwe (8.7 [95% CI: 7.0–10.9] versus 5.5 [95% CI: 4.7–6.6]) (Figure 2). Rural household with any PLWH had higher death rates compared to rural households without any PLWH in four countries: Tanzania (11.8 [95% CI: 9.7–14.3] versus 6.6 [95% CI: 6.0–7.2]), Malawi (9.5 [95% CI: 7.7–11.6] versus 5.5 [95% CI: 4.9–6.1]), Zambia (8.1 [95% CI: 6.5–10.1] versus 5.3 [95% CI: 4.8–5.9]), and Kenya (9.0 [95% CI: 7.2–11.6] versus 6.4 [95% CI: 5.9–7.0]).

**Figure 3:**
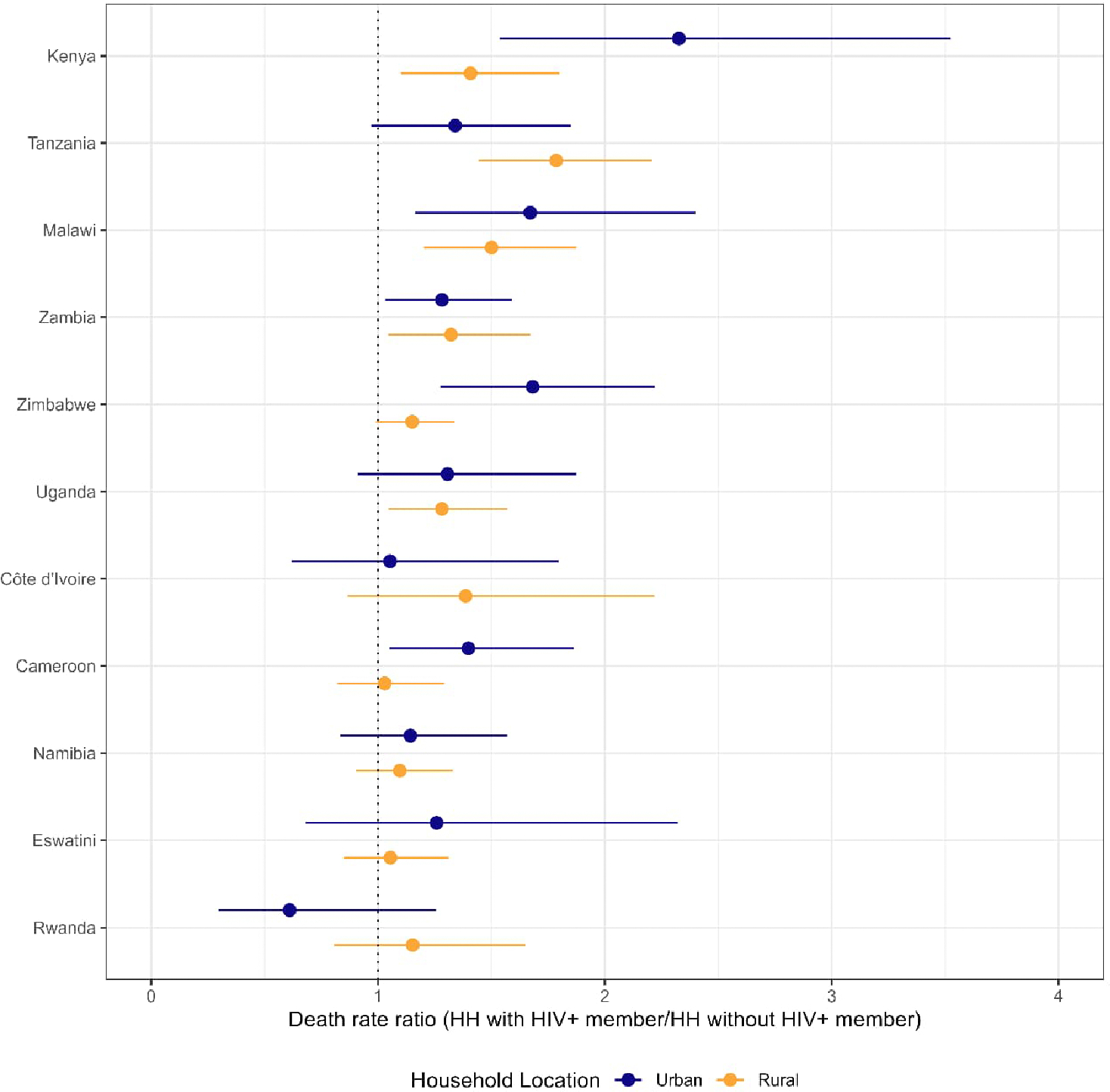
Death rate ratios comparing households with PLWH and households without PLWH by urban/rural, including all ages and both sexes combined in 11 countries in the three years before the survey, 2015–2019

Death rate ratios comparing households with PLWH versus those without PLWH were significantly greater than 1 for Kenya (urban 2.3 [95% CI: 1.5–3.5], rural 1.4 [95% CI: 1.1–1.8]), Tanzania (rural 1.8 [95% CI: 1.4–2.2]), Malawi (urban 1.7 [95% CI: 1.2–2.4], rural 1.5 [95% CI: 1.2–1.9], Zambia (urban 1.3 [95% CI: 1.03–1.6], rural 1.3 [95% CI: 1.04–1.7]), Zimbabwe (urban 1.7 [95% CI: 1.3–2.2]), Uganda (rural 1.3 [95% CI: 1.04–1.6]), and Cameroon (urban 1.4 [95% CI: 1.1–1.9]) (Figure 3).

## 4. Discussion

In this description of HIV-related excess mortality using deaths reported in households from 11 nationally representative general population surveys, we found that households with PLWH had generally higher death rates than those without PLWH. This was true overall, for males and females, and for most age groups. This pattern was present when death rates were stratified by urban versus rural HH location, although the urban/rural difference was not statistically significant in all countries. Collectively these findings—among households with PLWH overall and stratified by sex, age, and urban/rural—indicate that there may have been excess mortality among households with PLWH compared to those without PLWH, particularly in Kenya and Tanzania. ART has decreased HIV mortality over the past 20 years, but mortality is still higher among PLWH (Brinkhof et al. 2009; Slaymaker et al. 2014; Ghislain, Mushebenge, and Magula 2021; Gona et al. 2020). In SSA, overall mortality between 2009 and 2018 among PLWH remained three times higher than among people without HIV (Brinkhof et al. 2009; Slaymaker et al. 2014; Ghislain, Mushebenge, and Magula 2021; Gona et al. 2020). These findings and those from other studies suggest excess mortality among households with PLWH that extends beyond the direct impact of HIV on the health of the person living with HIV. When death rates were assessed by location, higher death rates were demonstrated among rural compared with urban households, with statistical significance in five country-specific comparisons, similar to reports from other countries (Menashe-Oren and Stecklov 2018). In contrast to our findings, other studies have identified an urban penalty, where death rates from all causes in some urban areas were higher than in rural areas due to worsening urban socioeconomics, infrastructure, and public health (Gould 1998; Rice and Rice 2009). We did not find evidence of an urban penalty.

Strengths of this analysis include the use of rigorously collected, nationally representative data from 11 countries and the objective verification of HIV status of all living members of each participating HH. Limitations include lack of information about causes of death and reliance on the HH respondent’s answers to five questions about HH deaths. Our finding of excess mortality associated with households with PLWH may be an underestimate, as households without PLWH may have lost members to HIV/AIDS before the three-year period of recall or the HH respondent may have forgotten about a death or felt too uncomfortable to report a death. Additionally, PHIA surveys did not include institutionalized individuals, a bias likely to underestimate death rates. However, the HH respondent may have included institutionalized individuals when reporting on deceased HH members. Finally, since children in only a subsample of households (33%–50%) were eligible for blood testing in any given survey, some HIV+ children may have been missed and their households may have been misclassified as not having an HIV+ household member. This would have only a minimal effect on results, however, because HIV prevalence among children in the surveyed countries was low and all but a handful of children living with HIV had mothers living with HIV (Reid et al. 2021).

This analysis did not measure indirect adverse effects of having PLWH as HH members, such as whether stigma and discrimination interfered with health care access or the impact of HIV illness on household resources. In addition, given the high prevalence of tuberculosis in all 11 countries, especially among PLWH, it is possible that households with PLWH were also more likely to have the adverse impact of having someone living with tuberculosis in the HH (World Health Organization 2018). The provision of preventive health services, such as TB screening, to households that include PLWH warrants further study. Most HIV care and treatment services are focused on individuals, but TB prevention studies have demonstrated the advantage of providing preventive services to household members (Zachariah et al. 2003).

## 5. Conclusion

These findings demonstrate the continued need for more country-specific data to assess mortality trends, as aggregate analyses may mask changing mortality patterns in individual countries. These findings suggest a new approach of using survey data to capture indirect effects of HIV on mortality of HH members and to track progress toward zero AIDS-related deaths. Future research may delineate whether the observed excess mortality in households with PLWH was due to indirect factors related to the impact of HIV on a household or was solely because PLWH experience higher mortality.

## Figures and Tables

**Table 1:** Household death rate, by country, for 11 countries during the three years before each PHIA survey, 2015-2019

	Country										
Characteristic*	Cameroon	Côte d’Ivoire	Eswatini	Kenya	Malawi	Namibia	Rwanda	Tanzania	Uganda	Zambia	Zimbabwe

	N	N	N	N	N	N	N	N	N	N	N
	
**Total household deaths**	1,767	729	459	1,267	891	951	613	1,359	1,125	1,077	1,208
**Urban**	559	287	61	415	226	331	113	368	204	375	259
**Rural**	1,208	442	391	852	498	620	500	991	921	516	880
**Total de jure household population**	54,553	39,270	20,512	65,090	44,658	39,094	53,419	67,667	62,759	50,859	46,787
**Urban**	21,669	18,944	3,631	21,735	13,869	15,336	11,901	20,094	15,188	18,830	11,928
**Rural**	32,884	20,326	16,381	43,355	25,278	23,758	41,518	47,573	47,571	27,138	32,515
**Percent HH with HIV+ member**											
**Urban**	7.4 (6.7, 8.2)	5.6 (4.8, 6.5)	41.7 (39.1, 44.5)	7.1 (6.4, 7.9)	22.4 (20.8, 24.1)	16.7 (15.4, 18.0)	10.6 (9.5, 11.9)	11.2 (10.2, 12.2)	16.3 (15.0, 17.7)	25.7 (24.4, 27.0)	23.5 (22.0, 25.0)
**Rural**	7.4 (6.6, 8.2)	3.8 (3.2, 4.6)	45.4 (43.8, 47.0)	8.4 (7.8, 9.0)	12.9 (12.1, 13.7)	25.5 (24.3, 26.8)	5.8 (5.3, 6.3)	8.3 (7.7, 9.0)	11.8 (11.1, 12.6)	13.4 (12.6, 14.3)	22.3 (21.4, 23.3)
**Overall**	7.4 (6.9, 7.9)	4.9 (4.3, 5.5)	44.2 (42.8, 45.6)	7.9 (7.4, 8.4)	14.5 (13.8, 15.2)	20.4 (19.5, 21.4)	6.7 (6.3, 7.2)	9.4 (8.9, 10.0)	13.1 (12.5, 13.8)	18.5 (17.7, 19.2)	22.7 (21.9, 23.5)
**1 HIV+ member**	6.5 (6.0, 7.0)	4.2 (3.7, 4.8)	31.0 (29.7, 32.3)	6.6 (6.1, 7.0)	11.2 (10.6, 11.9)	15.9 (15.1, 16.8)	5.3 (4.9, 5.7)	7.8 (7.3, 8.3)	10.4 (9.9, 11.1)	13.7 (13.0, 14.3)	16.7 (16.0, 17.4)
**2+ HIV+ members**	0.9 (0.8, 1.2)	0.7 (0.5, 0.9)	13.3 (12.4, 14.2)	1.3 (1.1, 1.6)	3.3 (2.9, 3.6)	4.5 (4.1,5.0)	1.5 (1.2, 1.7)	1.6 (1.4, 1.9)	2.7 (2.4, 3.0)	4.8 (4.4, 5.3)	6.0 (5.6, 6.5)
**Total responding households**	11623	8983	5185	16918	11386	9315	11219	14811	12386	10957	11717
**Households missing death data**	31 (0.3%)	16 (0.2%)	57 (1.1%)	22 (0.1%)	1 (0.0%)	85 (0.9%)	5 (0.0%)	19 (0.1%)	2 (0.0%)	9 (0.1%)	5 (0.0%)
**Households with no eligible adults**	416 (3.6%)	187 (2.1%)	0 (0.0%)	850 (5.0%)	817 (7.2%)	293 (3.1%)	190 (1.7%)	0 (0.0%)	305 (2.5%)	405 (3.7%)	0 (0.0%)
**Households with no valid HIV results**	543 (4.7%)	670 (7.5%)	259 (5.0%)	1407 (8.3%)	1261 (11.1%)	838 (9.0%)	58 (0.5%)	513 (3.5%)	152 (1.2%)	1211 (11.1%)	782 (6.7%)
**Households with at least one HIV result for an eligible adult**	1728 (14.9%)	2623 (29.2%)	1273 (24.6%)	5276 (31.2%)	3743 (32.9%)	3202 (34.4%)	2201 (19.6%)	3880 (26.2%)	2283 (18.4%)	4177 (38.1%)	3366 (28.7%)
**Households with HIV results for all eligible adults Percent population in HH with HIV+ member**	8905 (76.6%)	5487 (61.1%)	3596 (69.4%)	9363 (55.3%)	5564 (48.9%)	4897 (52.6%)	8765 (78.1%)	10399 (70.2%)	9644 (77.9%)	5155 (47.0%)	7564 (64.6%)
**Urban**	8.7 (8.3, 9.1)	6.5 (6.1, 6.9)	47.0 (45.4, 48.7)	8.2 (7.8, 8.6)	23.4 (22.5, 24.2)	20.5 (19.9, 21.3)	11.8 (11.2, 12.4)	11.8 (11.3, 12.4)	18.0 (17.4, 18.7)	26.4 (25.8, 27.0)	24.7 (24.0, 25.5)
**Rural**	8.5 (8.2, 8.9)	5.0 (4.6, 5.4)	50.0 (49.2, 50.8)	8.8 (8.5, 9.1)	13.3 (12.9, 13.7)	28.7 (28.2, 29.3)	6.0 (5.7, 6.2)	8.2 (7.9, 8.5)	12.0 (11.7, 12.4)	14.5 (14.1, 14.9)	23.3 (22.9, 23.8)
**Overall**	8.6 (8.4, 8.9)	5.8 (5.6, 6.1)	49.3 (48.6, 50.0)	8.6 (8.4, 8.8)	15.0 (14.7, 15.4)	24.6 (24.1, 25.1)	7.0 (6.8, 7.2)	9.4 (9.2, 9.7)	13.6 (13.3, 13.9)	19.3 (19.0, 19.7)	23.8 (23.4, 24.2)
**Estimated death rates per 1,000 person-years (95% CI)**	7.9 (7.5, 8.4)	5.9 (5.4, 6.5)	7.9 (7.1, 8.8)	6.5 (6.0, 7.0)	5.9 (5.4, 6.4)	5.8 (5.4, 6.3)	4.0 (3.7, 4.3)	6.7 (6.3, 7.2)	5.9 (5.6, 6.4)	5.8 (5.4, 6.3)	7.7 (7.3, 8.3)
**Urban**	6.5 (5.9, 7.1)	5.1 (4.4, 5.9)	5.7 (4.2, 7.9)	6.1 (5.3, 7.1)	4.7 (3.9, 5.6)	5.1 (4.4, 5.9)	3.2 (2.6, 3.9)	6.0 (5.3, 6.8)	4.4 (3.7, 5.1)	5.9 (5.3, 6.6)	6.4 (5.6, 7.3)
**Rural**	9.3 (8.7, 9.9)	7.0 (6.2, 7.9)	8.6 (7.7, 9.6)	6.6 (6.1, 7.2)	6.1 (5.5, 6.7)	6.6 (6.0, 7.2)	4.1 (3.8, 4.6)	7.1 (6.5, 7.7)	6.5 (6.0, 7.0)	5.8 (5.2, 6.3)	8.4 (7.8, 9.0)

*Notes*:

* Unweighted counts. “Total household deaths” is the sum across all households in each country of the number of individual deaths per HH reported by the HH respondent as occurring during the three years before each survey; estimated death rates were computed using quasi-Poisson regression incorporating jackknife survey weights.

**Table 2:** Household death rate and death rate ratios using pooled data across 11 countries by household PLWH status and by demographic characteristics, 2015–2019

	Death rate per 1,000 person-years (95% CI)			
Characteristic	Overall	In HH with PLWH	In HH without PLWH	Death rate ratio

**Age**				
0–5*	6.0 (5.6, 6.5)	6.4 (5.1, 8.0)	6.0 (5.5, 6.5)	1.1 (0.8, 1.4)
6–14	0.9 (0.8, 1.0)	1.3 (0.9, 1.8)	0.8 (0.7, 1.0)	**1.5 (1.1, 2.2)**
15–24	1.2 (1.1, 1.4)	1.5 (1.1, 2.0)	1.2 (1.0, 1.4)	1.2 (0.9, 1.7)
25–34	1.7 (1.5, 2.0)	3.4 (2.7, 4.3)	1.5 (1.3, 1.8)	**2.2 (1.7, 2.9)**
35–49	3.4 (3.1, 3.7)	5.1 (4.2, 6.3)	3.1 (2.7, 3.4)	**1.7 (1.3, 2.1)**
50–64	3.8 (3.3, 4.3)	3.1 (2.3, 4.3)	3.9 (3.4, 4.5)	0.8 (0.6, 1.1)
65+	11.2 (10.1, 12.4)	14.8 (11.6, 18.7)	10.8 (9.7, 12.1)	**1.4 (1.1, 1.8)**
**Sex**				
Male	6.4 (6.2, 6.7)	8.6 (7.8, 9.3)	6.1 (5.9, 6.4)	**1.4 (1.3, 1.5)**
Female	5.1 (4.9, 5.2)	7.0 (6.4, 7.8)	4.8 (4.6, 5.0)	**1.5 (1.3, 1.6)**
**Location**				
Urban	5.3 (5.1, 5.6)	7.3 (6.6, 8.2)	5.1 (4.8, 5.3)	**1.5 (1.3, 1.6)**
Rural	6.9 (6.7, 7.1)	9.2 (8.5, 9.9)	6.6 (6.4, 6.8)	**1.4 (1.3, 1.5)**
**Total**	6.3 (6.2, 6.5)	8.6 (8.1, 9.2)	6.0 (5.9, 6.2)	**1.4 (1.3, 1.5)**

*Note*:

* Ages of death for the youngest age group, 0–5, were all bottom coded to 5 during dataset preparation to protect confidentiality due to small counts.

## Data Availability

The data that support the findings of this study are available upon request from the PHIA project website: https://phia-data.icap.columbia.edu/.
